# Modifiable Factors for the Trajectory of Health-Related Quality of Life among Youth Growing Up in Poverty: A Prospective Cohort Study

**DOI:** 10.3390/ijerph18179221

**Published:** 2021-09-01

**Authors:** Ko Ling Chan, Camilla Kin Ming Lo, Frederick K. Ho, Qiqi Chen, Mengtong Chen, Patrick Ip

**Affiliations:** 1 Department of Applied Social Sciences, The Hong Kong Polytechnic University, Hung Hom, Hong Kong; camilla.lo@polyu.edu.hk (C.K.M.L.); jenna.mt.chen@polyu.edu.hk (M.C.); 2 Institute of Health and Wellbeing, University of Glasgow, Glasgow G12 8RZ, UK; Frederick.Ho@glasgow.ac.uk; 3 Department of Social Work, School of Sociology and Anthropology, Xiamen University, Xiamen 361005, China; qqchen@xmu.edu.cn; 4 Department of Paediatrics and Adolescent Medicine, LKS Faculty of Medicine, The University of Hong Kong, Pokfulam, Hong Kong; patricip@hku.hk

**Keywords:** poverty, child, health-related quality of life

## Abstract

Poverty is a decisive risk factor for poor health and well-being, and its negative consequences could be more severe and substantial among children. Understanding the factors associated with improvement in well-being is vital to design interventions. This is a prospective cohort study of 546 youth growing up in families in poverty in Hong Kong. All participants were assessed twice, in 2016 and 2019, in regard to their physical and mental health, as well as for different economic, social, and psychological variables. The results show that approximately 41% experienced an improvement in their health-related quality of life (HRQoL). Findings from the logistic regression analyses suggest that the health and development of youth in poverty may be restored by promoting social support, a sense of hope, future orientation, job stability, and money management practices, such as savings, during childhood and adolescence. The findings shed light on future policy making and forms of service development that could help to end the vicious cycle of poverty and hampered health.

## 1. Introduction

Poverty is one of the major risk factors leading to hampered health and well-being [1]. It has been shown that poverty can be associated with physical and mental health problems in a vicious cycle; furthermore, poverty is related to higher risks of mental disorders and poor physical health, which could lead to job instability and financial burdens [2,3]. Poverty could impose serious negative consequences on individuals and their families, including those living in developed countries, such as the United States, the United Kingdom, and Hong Kong [4,5,6].

Compared to adults living in poverty, children growing up in poverty may experience more severe chronic problems in regard to their health and development. The extant literature has provided plenty of evidence that children in poverty tend to have poorer physical, emotional, social, and educational outcomes, as well as higher risks of early death in the long term [5,7]. Poor health during the critical periods of childhood and adolescence may seriously hinder future development, which further limits future health and social positions in later life [8].

To end the vicious cycle of poverty and poor health, a better understanding of the mechanisms through which poverty affects health and well-being among children growing up in poverty is needed. However, not all children growing up in poverty share similar negative impacts on their health conditions. Some may experience more negative effects on their health conditions but others may be more resilient. Existing poverty reduction programs may target the alleviation of poverty through the development of human capital by the investment of education [9], early childhood education programs [10], or direct cash transfer [11,12]. 

Disadvantaged backgrounds, such as intergenerational poverty, disadvantaged neighbourhoods and lack of resources, may reinforce these perceptions and make it challenging to build up human capital [12]. Centred around the development of human capital, enhancing the academic and health-related outcomes of children in poverty would help the children to break the cycle of poverty. While the poverty reduction programs may effectively reduce poverty, they may not adequately address adverse health conditions. 

Focusing exclusively on poverty reduction might not be able to help alleviate health conditions. Elucidating the moderating and mediating factors of the associations between poverty and hampered health may help identify circumstances that are accountable for interventions. Our previous study has shown that interventions that address behavioural problems, social support, hope or future orientation, and study motivation are associated with a better quality of life for children living in poverty [13].

Social support is a significant factor that could facilitate healthy development among children living in poverty, particularly a better health-related quality of life (HRQoL) [6]. Some modifiable economic, sociological and psychological factors might lead to improved HRQoL, which can be hampered among children who grow up under the adverse impact of poverty [5].

Future orientation, a concept referring to one’s thoughts, plans, motivations, and feelings about the future [14], is of great importance to the identity development and commitment of children and adolescents [15]. A positive future orientation or hope motivates children to place greater values on future development and to be visionary of their expected future [16], which, in turn, influences their short-term actions and behaviours. Children in poverty may perceive their future as very distant and less hopeful. They may underestimate the value of investments in education and health-related practices, which can enhance their economic and health status [11].

Using longitudinal data obtained from a cohort of youth growing up in poverty, this study aimed to identify and examine the effects of changes in economic status, social, and psychological factors on changes in their HRQoL. In particular, we set out to identify alterable variables contributing to the improvement in HRQoL among youth growing up in poverty, to give insights into future policy making and intervention development. The study will provide unique data showing the factors that could alleviate health conditions impacted by poverty. It will help policymakers shape the poverty reduction programs by incorporating modifiable factors that could also benefit youth growing up in poverty.

## 2. Methods

### 2.1. Study Design and Sampling

This study employed cohort data from a longitudinal follow-up study of youth living in poverty in Hong Kong. A sample of school-aged youth from families living in poverty were recruited in 2016. When recruited, these families were receiving financial assistance from the Hong Kong government, including Comprehensive Social Security Assistance and full grants from Student Financial Assistance Schemes, or were those whose monthly household income was lower than 75% of the median monthly domestic household income, which was equivalent to 3205 US dollars (USD) [13]. 

All participants were assessed twice, in 2016 and 2019, in regard to their physical and mental health, as well as different economic, social, and psychological variables. Detailed descriptions of the longitudinal study have been published elsewhere [13,17]. All study procedures were approved by the Institutional Review Board of the University of Hong Kong/Hospital Authority Hong Kong West Cluster (Ref. No.: UW16-232).

The data used in this study were obtained from a cohort of 546 youth growing up in poverty (aged 17 to 27 years in 2019, mean = 20.6 years, *SD* = 2.7), who had provided complete records for the study. About 63% reported that they were students, while 37% were working for a living in 2019. On average, the monthly family gross income of the sample was approximately 2352 USD (*SD* = 1098) and the average income *per capita* was approximately 594 (*SD* = 344) USD. Details of the demographic characteristics of the overall sample are summarised in Table 1. A total of 46% of the sample were male, and there was no significant gender difference observed when we compared all the variables in this study.

### 2.2. Measures

#### 2.2.1. Outcome Measures

Improvement in the HRQoL of the sample was measured using the Chinese version of the 23-item Pediatric Quality of Life Inventory (PedsQL), Generic Core Scale [18]. The PedsQL comprises four subscales, measuring the HRQoL related to different aspects of life: physical functioning (eight items), emotional functioning (five items), social functioning (five items), and school functioning (five items). All items were rated on a five-point Likert scale, and the scores were summarised to provide a total score and four subscale scores. In this study, improvement in HRQoL was defined as any increase in the scores reported by a participant from 2016 to 2019. The outcome variable was recorded on a dichotomous basis (i.e., yes/no) to explore the direction of change, regardless of the magnitude of changes, as this could help to identify protective factors accountable for the improvement in HRQoL.

#### 2.2.2. Economic Status, Social, and Psychological Measures

Emotional and behavioural problems were assessed using 20 items from the Chinese version of the Strengths and Difficulties Questionnaire (SDQ), which covers conduct problems, emotional problems, hyperactivity, and peer problems [19]. All items were rated on a three-point scale, and the item scores were summed to produce a total score. The higher the score, the more severe the problems.

Hope related to one’s future was measured with the 12-item Hope Scale, which covers one’s goal-oriented determination and one’s plans to achieve one’s goals [20]. All items were rated on a four-point scale, and the item scores were summed to provide a total score. Higher scores indicated higher levels of perceived hope for one’s future.

Perceived social support was measured using the Chinese version of the 12-item Multidimensional Scale of Perceived Social Support (MSPSS), which captures one’s social support from family, friends, and significant others [21]. All items were rated on a seven-point scale; the higher the score, the higher the level of perceived social support.

Future orientation in regard to one’s education and career was assessed with the two 14-item subscales of the Chinese version of the Exploration and Commitment Questionnaire [22]. All items were rated on a five-point scale. Higher scores indicated greater commitment to one’s future education or future career and, thus, a more positive orientation in regard to one’s future.

In this study, an increase or a reduction in emotional and behavioural problems, hope, social support, and future orientation regarding education and career was defined as any change in the scale score of the participant from 2016 to 2019. The variables were coded in a dichotomous manner (i.e., yes/no).

Economic variables in this study included whether or not the participants had a savings practice and had received any form of social security assistance from the government in 2019. The participants’ economic activity status (i.e., whether or not they were working for a living or studying at schools/universities) and their monthly family gross income in 2019 were also recorded.

Demographic characteristics, including age, gender, and education attainment in 2019, were also included for analysis in this study.

### 2.3. Statistical analysis

All statistical analyses were conducted using SPSS 25.0 (IBM Corp., Armonk, NY, USA). All *p*-values were two-tailed and the significance level was set as 0.05. The outcome variables and other variables were summarised with descriptive statistics. Using F-statistics and chi-square tests, all variables were compared between the group of participants with improvements in HRQoL and the group of participants without. 

The demographic, economic status, social, and psychological correlates were examined using a structured multiphase binary logistic regression analysis, with improvements in HRQoL as the dependent variable. This is based on the assumption that two phases of variables have a sequential causal relationship but not vice versa, like demographic variables may affect psycho-social behaviours but not vice versa. For each dependent variable, we first conducted a forward stepwise logistic regression on all demographic characteristics, as well as the economic variables in Phase 1. 

Then, we included each of the social and psychological variables in the regression models, with adjustments for the variables in Phase 1 and other variables in Phase 2. We considered *p* < 0.05 as statistically significant. The Hosmer and Lemeshow (H–L) test was used to calculate the model fit for logistic regression models [23]. Missing data were handled with listwise deletion in all analyses. Multicollinearity was checked to ensure the independent variables were not strongly inter-correlated.

## 3. Results

Table 1 summarises the descriptive statistics in this study. About 52% of the sample had a savings practice. Concerning the changes in psychological variables from 2016 to 2019 among the 546 adolescents, 73% reported a reduction in emotional and behavioural problems, 28% had an increase in hope in regard to their future, 23% had an increase in their perceived level of social support, 35% reported an increase in future orientation regarding education, and 34% reported an increase in future orientation related to their career.

Of the 546 youth in this study, 41% (*n* = 223) reported an improvement in their HRQoL from 2016 to 2019. The group with improvements in regard to HRQoL (*n* = 223) and the group with no improvement in regard to HRQoL (*n* = 323) were compared in regard to their demographic characteristics, economic, social, and psychological variables, and psychological variables. 

Significant between-group differences were observed across age, education attainment, economic activity status, family income, the obtaining of financial assistance from the government, a savings practice, hope, social support, and future orientation regarding one’s career. Specifically, youth with improvements in regard to HRQoL tended to be of an older age, with higher education attainment, working for a living, a higher family income, were less likely to receive financial assistance from the government, and had a savings practice, improved hope regarding the future, improved social support, and improved future orientation regarding their careers.

Table 2 shows the results of the two-phase logistic regression analyses, after the adjustment for possible confounding variables. Regarding the overall HRQoL, working for a living compared with being a full-time student was associated with a higher likelihood that an improvement in HRQoL occurred (adjusted *OR* = 9.85 [95% *CI* = 4.76–20.40], *p* < 0.001). In addition, reduced emotional and behavioural problems (*aOR* = 1.95 [1.10–3.45], *p* < 0.05), increased hope (*aOR* = 2.89 [1.67–5.01], *p* < 0.001), and increased social support (*aOR* = 2.28 [1.31–3.98], *p* < 0.01) were associated with a higher likelihood of improved HRQoL.

When breaking down HRQoL into its four aspects, each of the four aspects was found to have different associations with the demographic characteristics, economic, social, and psychological variables. Improved physical functioning was found to be associated with savings practice (*aOR* = 2.27 [1.32–3.93], *p* < 0.01), increased hope (*aOR* = 2.68 [1.55–4.66], *p* < 0.001), and increased social support (*aOR* = 1.94 [1.11–3.40], *p* < 0.05). Improved emotional functioning was associated with working for a living (*aOR* = 3.67 [1.74–7.75], *p* < 0.001), reduced emotional and behavioural problems (*aOR* = 2.54 [1.32–4.87], *p* < 0.01), increased hope (*aOR* = 2.33 [1.36–4.00], *p* < 0.01), increased social support (*aOR* = 1.94 [1.13–3.94], *p* < 0.05), and increased future orientation regarding one’s career (*aOR* = 1.95 [1.12–3.39], *p* < 0.05). 

Increased social functioning was associated with working for a living (*aOR* = 2.26 [1.03–4.99], *p* < 0.05), receiving financial assistance from the government (*aOR* = 2.45 [1.17–5.14], *p* < 0.05), experiencing increased hope (*aOR* = 2.17 [1.22–3.87], *p* < 0.01), increased social support (*aOR* = 3.48 [1.97–6.13], *p* < 0.001), and increased future orientation regarding one’s career (*aOR* = 2.06 [1.13–3.75], *p* < 0.05). Finally, improved school functioning was associated with older age (*aOR* = 1.61 [1.42–1.83], *p* < 0.001), receiving financial assistance from the government (*aOR* = 3.99 [2.03–7.83], *p* < 0.001), increased hope (*aOR* = 3.66 [1.99–6.72], *p* < 0.001), increased social support (*aOR* = 2.10 [1.14–3.89], *p* < 0.05), and increased future orientation regarding one’s career (*aOR* = 2.46 [1.37–4.40], *p* < 0.01).

## 4. Discussion

Using the longitudinal data of a cohort of youth growing up in poverty in Hong Kong, we demonstrated that several demographic characteristics, including economic, social, and psychological variables, could be associated with an improvement in HRQoL. The results from the adjusted analyses of the longitudinal data indicated that impoverished youth who reported working for a living, who showed reduced emotional and behavioural problems, who perceived an increased level of hope regarding their future, and who had experienced improved social support when compared to their past were more likely to report improvements in their HRQoL. 

The findings showed that youth living poverty might not necessarily grow up under the toxic impact of poverty and related adverse childhood experiences [5]. Instead, some economic and psychological factors or outcomes might be modifiable in the long-term, which might, in turn, lead to an improved HRQoL, which had been hampered in childhood.

Social support has been identified in the literature as one of the most powerful factors that can promote healthy development among children [24]. Consistent with past studies [6,25,26], we found that improved social support perceived by children who had grown up in families living in poverty was associated with a better HRQoL. Social support, which is the presence of enduring relationships that help individuals develop a sense of security and interpersonal commitment [27], might play a role in promoting resilience and diminishing the negative influences of stress on developmental and adjustment outcomes among individuals living in adverse and stressful conditions. 

Indeed, there is plenty of evidence supporting the positive moderating and mediating effects of strong social support on the reduction of the negative impacts of poverty and related stress on one’s health and well-being [24]. This study extends previous knowledge by showing that improvements in perceived social support during adolescence could be associated with improvements in all aspects of HRQoL, including those related to physical, emotional, social, and school functioning, among children growing up in poverty. These results reflect the possibility of future interventions including components that could enhance the social support and social networks of children living in poverty, to help their development in the long term.

An improvement in the participants’ sense of hope, which was conceptualised as one’s determination and plans to achieve future goals [20], was found to be another psychological variable associated with enhanced HRQoL in all aspects among children growing up in poverty. This finding was in line with the results of a recent Filipino study, in which hope was demonstrated as a moderator of the association between financial stress and life satisfaction among young adults [28]. 

It has long been suggested in positive psychology that hope could buffer the impact of adverse experiences on well-being and lead to better coping strategies and a greater sense of happiness, in both direct and indirect ways [28,29,30,31]. In the case of children growing up in poverty, a greater sense of hope may lead to greater chances of using effective strategies to cope with stress related to poverty, such as asking for assistance or support from others, as hope could help children to make concrete plans to pursue future goals [28].

Compared with the other psychological variables included in this study, future orientation might be associated with HRQoL in different ways among children growing up in poverty. Future orientation regarding education was not related to any aspect of HRQoL in this study, while future orientation regarding one’s career was associated with QoL related to social and school functioning only. Together with the reduction in emotional and behavioural problems observed among children with improved HRQoL, this study is thus in line with previous research showing that a more positive future orientation among children might lead to fewer behavioural problems, better school performance, and enhanced health and well-being [32]. 

Future-oriented individuals tend to be more sensitive when considering the future consequences of their present behaviours when they make decisions, and thus they are less likely to experience behavioural problems, as they can see that demonstrating problematic behaviours may risk their future [33]. In this case, a positive future orientation regarding one’s career might motivate young adults to work hard to strive for better performance in school and encourage them to develop better social networks in order to prepare for their future, which might lead to better social and school functioning.

Concerning the economic variables related to HRQoL, this study revealed interesting results: Working for a living was associated with improved emotional and social functioning, while a savings practice was associated only with physical functioning. Compared with being a student, leaving school and working for a living might help to expand young adults’ social networks and broaden their horizons, which could, in turn, promote their HRQoL related to social functioning. 

In addition, job stability and job satisfaction might contribute, both directly and indirectly, to individuals’ subjective emotional HRQoL [34,35]. Job stability may lead to economic stability, especially among individuals coming from families living in poverty. Relief in regard to financial burden may then contribute to better emotional HRQoL via the reduction in stress. A savings practice, on the other hand, was associated only with the physical aspect of HRQoL. 

One possible explanation for this finding could be that having savings could make one more willing to spend money on medical expenses, or make it easier to gain access to better medical services when in need, thus leading to better subjective HRQoL related to physical functioning. Indeed, there is evidence that a savings practice is associated with satisfaction with HRQoL among adults [36]. The findings of this study may extend current knowledge by adding a piece of supportive evidence to the association between savings and physical HRQoL, which might provide important insights into future interventions for children living in poverty, to promote their physical health and well-being.

### Strengths and Limitations

The major strength of this study is its use of longitudinal data from a large cohort of youth growing up in poverty in Hong Kong. The longitudinal data enabled the tracking of HRQoL and potential correlates. This is similar to a difference-in-difference quasi-experimental design, minimising reverse causation to more robust results. Focusing on children growing up in poverty could also highlight their potential needs as previous research on modifiable factors of HRQoL often samples from general populations. This may skew toward children from families of better socioeconomic status.

On the other hand, this study has some limitations. First, data were collected via self-reports, and thus reporting biases may be present even though all instruments used have been validated in the local population. Second, the data only included a sample from Hong Kong, a Chinese society. The findings may, therefore, not be generalizable to other populations with different socioeconomic contexts. The outcome measures are coded dichotomously. This does not indicate the magnitude of improvement. 

The final model accounted for 36% of the improvement in the overall HRQoL. There may be other possible variables that could explain the changes in HRQoL in the sample, for instance, changes in socio-economic status of the whole family, health, or social services received by the participants. Future research may include other variables to explore more explanations for the extent of improvements in HRQoL among children living in poverty.

## 5. Conclusions

Poverty and its related adverse experiences could put individuals at high risk of hampered health and well-being. It is of great importance to understand what we can do to minimise the possible negative consequences of poverty on children’s health in the long term and how it is best to do so. This study provided preliminary evidence on the effects of social support, hope, future orientation, job stability, and money management practices, such as having a savings practice, on the improvement in HRQoL among individuals growing up in poverty. 

Future interventions should focus on poverty reduction and address the comorbidity of adverse health conditions among children and youth living in poverty. The intervention may include career and study guidance that could facilitate positive engagement in work and study and enhance optimistic hope and future orientation. Psychosocial interventions may be necessary to help reduce emotional and behavioural problems and to strengthen social support. If the interventions are developed, future research should examine the effectiveness of the intervention using experimental design. 

The findings can inform stakeholders in policy making in Hong Kong and shed light on the development of Child Development Fund (CDF), which is a community intervention that consists of encouraging a savings practice, mentorship components, and a personal development plan. The CDF encourages participating families to set a goal of saving HK$200 monthly over a 24 month period. Corporate donors and the Government contribute the same amount of matching grant for the savings. The savings can be used by the adolescents to implement their personal development plan with the guidance of their mentors. This poverty reduction program has proven to be effective in restoring the healthy development of children living in poverty in the future.

## Figures and Tables

**Table 1:** Summary of demographic characteristics and other financial and psychological variables by group of participants.

Variables	Total	Group without Improvement in HRQoL	Group with Improvement in HRQoL	*F*/chi-Square ^a^	*p*-Value
No. (%) with data	546 (100)	323 (59)	223 (41)	N/A	N/A
Age, mean (SD), year	20.6 (2.7)	19.8 (2.6)	21.8 (2.5)	−9.14	<0.001
Gender, No. (%)				2.31	0.13
Female	297 (54)	167 (52)	130 (58)		
Male	249 (46)	156 (48)	93 (42)		
Education attainment, No. (%)				22.09	<0.001
Secondary/high school or below	156 (29)	116 (36)	40 (18)		
Diploma, higher diploma, or associate degree	201 (37)	112 (35)	89 (40)		
University or above	189 (34)	95 (29)	94 (42)		
Economic activity status, No. (%)				128.96	<0.01
Student	345 (63)	267 (83)	78 (35)		
Working for a living	201 (37)	56 (17)	145 (65)		
Monthly family gross income, mean (SD), USD	2352 (1098)	2251 (1023)	2533 (1173)	−2.84	0.004
Recipient of any form of financial assistance from the government, No. (%)	173 (32)	124 (38)	49 (22)	16.43	<0.001
Savings practice, No. (%)	286 (52)	149 (46)	137 (61)	12.39	<0.001
Reduced emotional and behavioural difficulties, No. (%) ^b^	401 (73)	230 (71)	171 (77)	2.03	0.16
Increased hope, No. (%) ^b^	152 (28)	59 (18)	93 (42)	36.07	<0.001
Increased social support, No. (%) ^b^	126 (23)	52 (16)	74 (33)	21.69	<0.001
Increased future orientation regarding education, No. (%) ^b^	193 (35)	106 (33)	87 (39)	2.22	0.14
Increased future orientation regarding one’s career, No. (%) ^b^	183 (34)	82 (25)	101 (45)	23.46	<0.001

Note. HRQoL = health-related quality of life, as measured by the Pediatric Quality of Life Inventory (PedsQL). ^a^ F-tests or chi-square tests were conducted to compare the variables between the group with improvements in PedsQL and that without improvements in PedsQL, with the adjustment of the baseline data. ^b^ Scores were compared with the baseline data of each of the participants.

**Table 2:** Adjusted odds ratios (and 95% confidence intervals) of the two-stage logistic regression analyses using improvements in health-related quality of life as criteria.

Variable	Improved HRQoL (Overall)	Improved Physical Functioning ^a^	Improved Emotional Functioning ^a^	Improved Social Functioning ^a^	Improved School Functioning ^a^
Phase 1 ^d^					
Age	1.00 (0.88–1.13)	1.02 (0.89–1.18)	0.96 (0.84–1.10)	0.92 (0.79–1.06)	1.61 (1.42–1.83) ***
Gender (male)	0.95 (0.59–1.51)	0.89 (0.53–1.50)	0.86 (0.52–1.41)	1.27 (0.74–2.18)	1.13 (0.67–1.92)
Economic activity status (working for a living)	9.85 (4.76–20.40) ***	1.72 (0.81–3.65)	3.67 (1.74–7.75) ***	2.26 (1.03–4.99) *	N.A ^c^
Monthly gross family income (USD)	1.00 (1.00–1.00)	1.00 (1.00–1.00)	1.00 (1.00–1.00)	1.00 (1.00–1.00)	1.00 (1.00–1.00)
Recipient of any form of financial assistance from the government	1.17 (0.66–2.06)	1.62 (0.82–3.21)	1.15 (0.61–2.17)	2.45 (1.17–5.14) *	3.99 (2.03–7.83) ***
Savings practice	1.31 (0.81–2.11)	2.27 (1.32–3.93) **	1.14 (0.68–1.90)	0.88 (0.50–1.55)	1.48 (0.86–2.56)
Phase 2 ^d^					
Reduced emotional and behavioural difficulties ^b^	1.95 (1.10–3.45) *	1.63 (0.85–3.11)	2.54 (1.32–4.87) **	1.57 (0.80–3.08)	0.65 (0.36–1.19)
Increased hope ^b^	2.89 (1.67–5.01) ***	2.68 (1.55–4.66) ***	2.33 (1.36–4.00) **	2.17 (1.22–3.87) **	3.66 (1.99–6.72) ***
Increased social support ^b^	2.28 (1.31–3.98) **	1.94 (1.11–3.40) *	1.94 (1.13–3.94) *	3.48 (1.97–6.13) ***	2.10 (1.14–3.89) *
Increased future orientation regarding education ^b^	0.79 (0.47–1.35)	1.14 (0.66–1.96)	1.17 (0.69–2.00)	1.01 (0.57–1.80)	0.60 (0.33–1.10)
Increased future orientation regarding one’s career ^b^	1.27 (0.73–2.21)	1.48 (0.83–2.63)	1.95 (1.12–3.39) *	2.06 (1.13–3.75) *	2.46 (1.37–4.40) **
N	546	546	546	546	546
Nagelkerke R^2^	36%	24%	26%	25%	51%

Note. * *p* < 0.05. ** *p* < 0.01. *** *p* < 0.001. HRQoL= health-related quality of life, as measured by the Pediatric Quality of Life Inventory (PedsQL). Odds ratios were adjusted with the baseline data and other variables, apart from the specific independent variable and the criteria tested. Referent group: gender (female); economic activity status (students not working for a living); not a recipient of financial assistance from the government; no savings practice; unchanged or increased emotional and behavioural difficulties; unchanged or reduced hope; unchanged or reduced social support; unchanged or reduced future orientation regarding education; and unchanged or reduced future orientation regarding one’s career. ^a^ Physical functioning, emotional functioning, social functioning, and school functioning were captured using the four subscales of the Pediatric Quality of Life Inventory. ^b^ Scores were compared with the baseline data of each of the participants. ^c^ We did not perform a logistic regression in regard to school functioning with economic activity status as an independent variable because one of the groups did not consist of students and did not report data on school functioning. ^d^ Variables in phase 1 were adjusted by other variables in the same phase; variables in phase 2 were adjusted by all variables in phase 1 and other variables in phase 2.

## Data Availability

The data presented in this study are available on request from the corresponding author.
